# *SOFIA*: a knowledge-infused prompt-based reasoning framework for anxiety screening in social media

**DOI:** 10.3389/fdgth.2026.1899582

**Published:** 2026-08-14

**Authors:** Ashala Senanayake, Prasan Yapa, Sidath R. Liyanage

**Affiliations:** 1 Tamai Investment Education Inc, Kyoto, Japan; 2 Luxembourg Centre for Systems Biomedicine (LCSB), University of Luxembourg, Esch-sur-Alzette, Luxembourg; 3 Faculty of Computing & Technology, University of Kelaniya, Kelaniya, Sri Lanka

**Keywords:** anxiety screening, digital mental health, knowledge-enhanced PLMs, natural language processing, prompt-based reasoning

## Abstract

Linguistic-based anxiety screening has become a widely adopted approach for detecting anxiety in social media, with pre-trained language models (PLMs) now forming the state-of-the-art foundation for early mental health detection. However, most existing PLMs are not explicitly optimized for anxiety detection (AD), limiting their adaptability to fine-grained psycholinguistic cues. To address this gap, we introduce *SOFIA* (*Anxious? SOFten-It-Out*), a novel prompt-based reasoning framework that enhances PLM capability for binary AD without full fine-tuning. *SOFIA* integrates multiple prompt template strategies with a dual-end truncation method that preserves both initial contextual cues and concluding emotional markers, enabling richer and more stable representations of conversational anxiety signals. Additionally, a knowledge-enhanced verbalizer incorporates structured domain knowledge through soft, manual, and automatic verbalizers, improving reasoning within frozen PLMs. We evaluate *SOFIA* across three self-reported anxiety corpora, such as Guo, SMHD, and Kim, using multiple 100M−300M-parameter PLMs. Experimental results demonstrate consistent improvements over traditional baselines, including F1 score gains of 5.37%, 5.40%, and 7.92% for MentalBERT on the respective datasets when using soft templates of length 20 with knowledge base-driven verbalizers. These findings highlight *SOFIA*'s effectiveness as a scalable, low-resource, and knowledge-informed framework for digital mental health screening applications.

## 1 Introduction

According to Statista, more than half of the global population actively uses social media. The COVID-19 pandemic further accelerated this trend, as lockdowns and disruptions to daily life pushed people to rely heavily on digital platforms for communication, entertainment, and social connection. This surge in usage led to an exponential rise in the sharing of personal content, ranging from life updates such as birthdays, anniversaries, and travel experiences to professional milestones, photos, videos, invitations, and expressions of opinions or emotions. Social media thus became a vast repository of human thoughts and feelings, often serving as a mirror of individuals' psychological states (1). The frequency and nature of shared content can provide valuable insights into underlying mental health conditions, including anxiety. Yet nearly 80% of individuals delay or avoid seeking treatment, which underscores the value of social media expressions as an indirect indicator of psychological wellbeing (2). A statistical survey conducted in the United Arab Emirates in 2018 reported prevalence rates of 55% for anxiety, 38% for depression, and 29% for stress related disorders (3). Comparable patterns were found in other populations. Among Turkish university students, the rates were 47.1% for anxiety, 27.1% for depression, and 27% for stress (4). A study of tertiary students in Hong Kong reported 21%, 41%, and 27%, respectively (5). Taken together, these findings show that anxiety is particularly common when compared to other emotional disturbances across different cultural and educational settings.

Linguistic based anxiety screening has therefore emerged as one of the most widely used approaches for detecting anxiety in social media. Unlike emotion-based or vision-based methods, it focuses on analyzing the language patterns, word choices, and contextual cues in users' posts to identify signs of psychological distress (6, 7). Linguistic methods are often more effective because language can capture subtle cognitive and emotional markers that may not be evident in facial expressions or visual content, offering a richer and more reliable representation of a person's mental state. Within this domain, approaches such as sentiment analysis (8, 9), topic modeling (10), linguistic feature analysis (11), and machine learning techniques (12, 13) leverage natural language processing (NLP) signals to support downstream anxiety detection. More recently, pre-trained language models (PLMs) have advanced linguistic-based anxiety screening by enabling deeper contextual understanding, allowing subtle cues in word choice, phrasing, and contextual patterns to be detected, thereby improving the accuracy of mental health assessment from online text (14, 15). However, most existing PLMs are not tailored specifically for anxiety, even though specialized models have been developed for related conditions such as depression (16). This highlights the need for more targeted approaches, as non-fine-tuned methods are often better suited for settings with limited annotated data and diverse task requirements.

Prompt-based engineering techniques, on the other hand, have become increasingly popular as an alternative to full fine-tuning of PLMs (17). By reformulating downstream tasks as natural language prompts and a few examples, these methods allow models to adapt more flexibly to both contextually similar and distinct tasks without extensive retraining (18, 19). This approach has been particularly valuable in mental health applications, including few-shot depression screening (20), where data scarcity and task diversity make prompt-based methods more efficient than traditional fine-tuning. However, prompt-based methods have been applied to only a limited set of mental health tasks, and satisfactory progress has yet to be achieved in anxiety screening. This gap highlights the need for further research to fully leverage the potential of PLMs for detecting anxiety in social media contexts.

In this study, we propose *SOFIA* (*Anxious? SOFten-It-out*), a novel prompt-based framework designed to adapt mid-sized PLMs for anxiety detection. The proposed framework leverages multiple prompt templates to capture diverse linguistic cues and enhance model adaptability across different contexts. A knowledge base is also integrated into the framework to enhance reasoning and provide more transparent insights into the model's predictions. Our main contributions include: (1) a novel framework for adapting PLMs to mental health screening tasks by modifying the OpenPrompt library, enabling the transfer of large models to contextually different tasks by freezing most model parameters, inspired by recent prompt based tuning methods (21); (2) crafting prompt templates specifically tailored for downstream anxiety screening, while leveraging and improving truncation methods to ensure that the most relevant input signals are preserved for effective PLM prompting; (3) introducing a novel knowledge base that incorporates clinical domain knowledge to support and improve the reasoning capabilities of the model, thereby enhancing clinical relevance of its output; (4) evaluating downstream tasks to assess the generalization and robustness of the proposed methods across multiple benchmark corpora; and (5) conducting ablation studies to analyze decision mechanisms, explore the local decision space, and ensure the correctness of parameter choices.

The overall organization of this article is as follows: Section 2 reviews related work and Section 3 elaborates the proposed framework and methodology. Experiments are presented in Section 4 and then discussed in Section 5.

## 2 Related work

### 2.1 Linguistic-based anxiety detection

Research on linguistic-based anxiety detection has increasingly focused on analyzing textual signals from social media and online communication, aiming to uncover patterns that reflect underlying psychological states. Earlier approaches primarily relied on techniques such as n-gram analysis, linguistic inquiry and word count (LIWC), and sentiment analysis (22). However, because these methods capture only shallow or less quantifiable linguistic signals for mental health screening, their performance has generally been limited. Topic modeling approaches, such as latent Dirichlet allocation (LDA) and supervised LDA (23, 24), along with advanced language-based sentiment analysis techniques (25), have achieved moderate success on longer Reddit posts by providing richer contextual signals for downstream anxiety classification. Word embedding techniques (26, 27) were subsequently employed to capture semantic relationships between words, offering significant improvements in anxiety screening performance over traditional approaches. Building on this foundation, deep learning methods such as LSTMs (28, 29) and later attention-based models (30, 31) leveraged these embeddings to capture contextual dependencies more effectively, leading to further advances in linguistic-based risk detection for mental health. The introduction of PLMs, powered by self-attention mechanisms (32), marked a turning point by elevating NLP-based mental health screening to a significantly higher level of accuracy and robustness. Notable Bidirectional encoder representations from transformers (BERT)-based models in the mental health domain include PsychBERT (15), which is pre-trained on mental health and social media datasets to capture domain-specific linguistic patterns; MentalBERT (14), specifically customized for mental healthcare applications such as depression screening; and ClinicalBERT (33), fine-tuned for tasks including suicide risk detection. Beyond these, larger PLMs such as MentaLLaMA and Mental-Alpaca (34) have demonstrated strong performance in screening for depression and suicidal thoughts, leveraging their increased capacity to model complex contextual relationships in text and capture subtle indicators of psychological distress. However, despite their success in detecting depression and suicidal thoughts, none of these PLMs have been specifically developed or optimized for anxiety detection, leaving a significant gap for exploring more cost-effective alternatives to traditional fine-tuned customization methods.

### 2.2 Prompt-based methods for mental health

To address the limitations of traditional fine-tuning and the lack of anxiety-specific models, recent research has increasingly turned to prompt-based methods, which offer a flexible and cost-effective approach for adapting PLMs to mental health tasks. Recent research highlights that prompt engineering for mental health can be broadly categorized into four main approaches: vanilla prompting, in-context learning, prompt tuning, and instruction-based prompt tuning (35). Among these, zero-shot vanilla prompting with large PLMs, such as ChatGPT, has demonstrated strong capability for depression and suicide detection, though it is often considered computationally expensive and resource-intensive due to the reliance on large models (36–38). In-context learning approaches, such as the use of carefully chosen examples for depression detection (39), the exploration of mental health reasoning capabilities in large models (40), response generation techniques that enhance the explainability of ChatGPT and T5 models in mental health contexts (41), and conversational agents designed as therapy tools (42), extend the core idea by leveraging a few labeled examples within the prompt itself, thereby enabling frozen PLMs to adapt more effectively to mental health screening tasks without explicit fine-tuning. Prompt tuning, on the other hand, provides a cost-efficient and scalable alternative by adapting only a small set of parameters while keeping the majority of the PLM frozen. Notable applications include few-shot prompt-tuning-based domain transfer techniques in the mental health context (43), few-shot reinforcement learning frameworks for mental health-related generation tasks (44), evaluation protocols for temporal stress-level data (45), prompt-based retrieval-augmented generation and chain-of-thought prompting for tasks such as suicidal ideation detection (46, 47) and cognitive distortion identification (48). However, prompt tuning faces notable challenges, particularly with optimization stability and limited generalization to previously unseen tasks, which has motivated the development of instruction-based prompt tuning as a more flexible and adaptive alternative. Studies such as depression screening using contextually dissimilar knowledge transfer methods (49), NLP-based suicidal risk detection (50), and dialogue-based cross-task generalization for stress screening (51) have pioneered this category, yet their applications remain limited to a narrow set of mental health tasks. Recently, AnxiBERT by Senanayake and Liyanage (52) marked the first attempt at applying a prompt-tuning-driven method for anxiety detection using online data, though its scope was limited to basic PLM prompting without deeper reasoning capabilities. This highlights the need for more advanced prompt-based approaches that integrate contextual reasoning and domain knowledge to fully leverage the potential of PLMs in anxiety screening.

## 3 Methods

### 3.1 Problem formulation

We formulate anxiety detection as a prompt-based classification task. Let *D*= $\{\left(x_{i},y_{i}\right){\}}_{i=1}^{N}$ denote a dataset consisting of *N* textual samples, where $x_{i}$ ∈ *χ* represents a social media post as an input text and $y_{i}$ ∈ {0, 1} is the binary label indicating the presence (1) or absence (0) of anxiety-related signals. Each input $x_{i}$ is reformulated through a template *T*(⋅) into a prompted input $x_{i}^{\prime }$. A frozen PLM $M_{\theta }$ then predicts a verbalizer token v(y) corresponding to a label *y*. The verbalizer functions as a knowledge base, incorporating clinical domain knowledge to support downstream reasoning. The final prediction is obtained by maximizing $P_{\theta }(v(y)|x_{i}^{\prime })$, with training guided by minimizing the negative log-likelihood and keeping most parameters θ fixed, enabling efficient adaptation without full parameter tuning.

### 3.2 System design

**Figure 1:**
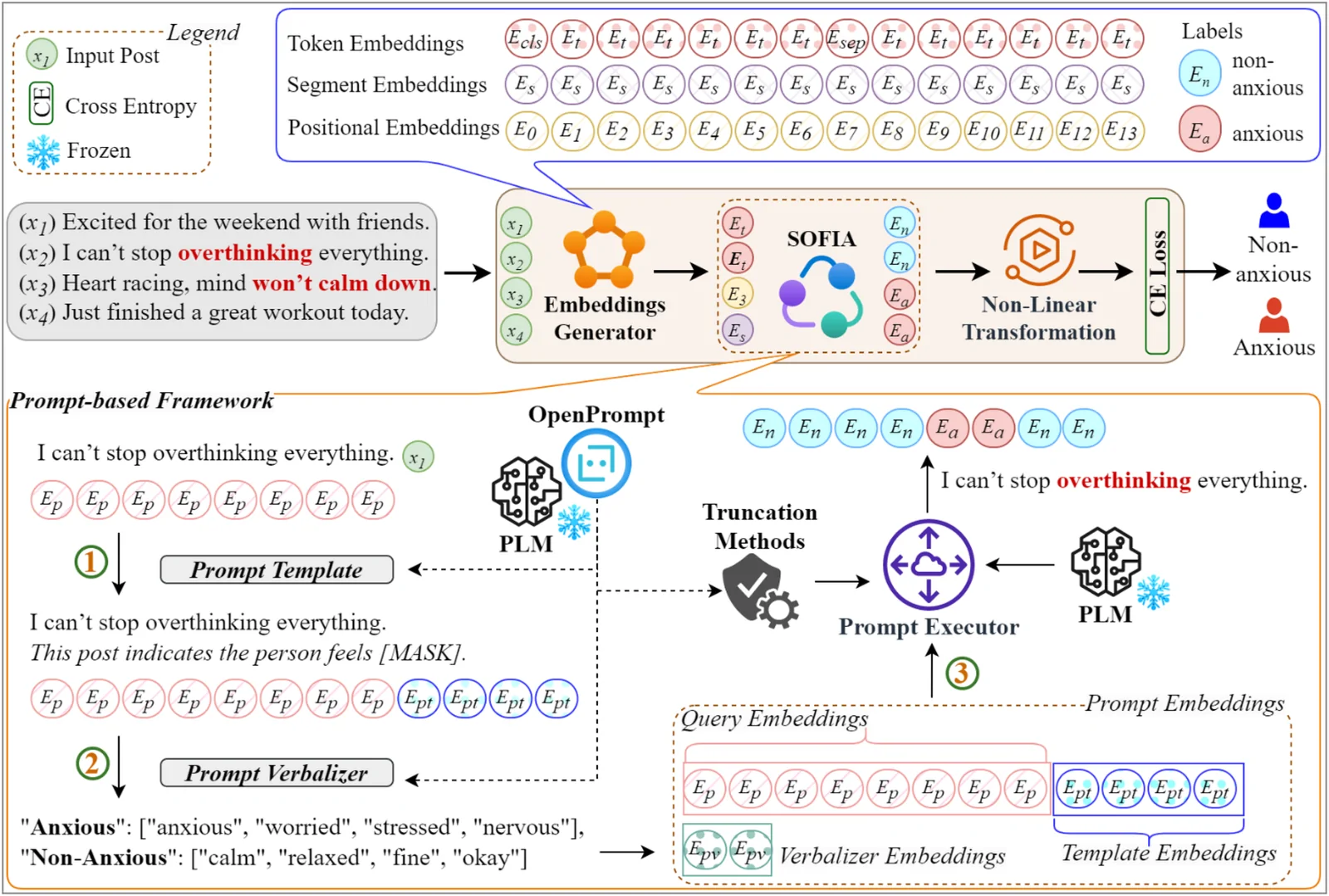
High-level system design of the proposed framework for anxiety detection.

The proposed system, shown in Figure 1, consists of three main components: embeddings generation, prompt-based framework, and downstream tasks formation.

### 3.3 Embeddings generation

In the proposed framework, feature extraction is performed using the same vanilla PLM *M* that is later employed for prompt-based downstream anxiety detection, serving as the encoder without fine-tuning its parameters. Given an input social media post $x_{i}$, the text is tokenized and augmented with special tokens such as [CLS] (classification token), [SEP] (segment separator), and [EOS] (end of sequence) where appropriate. Each token is then mapped into three contextualized embeddings: *token embeddings*, which capture semantic meaning of words; *segment embeddings*, which distinguish discourse units; and *positional embeddings*, which encode sequential order. The [CLS] token embedding serves as a fixed-dimensional representation of the entire post. A binary anxiety label $y_{i}$ ∈ {0, 1} is associated with each input $x_{i}$. These contextual embeddings capture rich semantic and syntactic information, and the resulting fixed-dimensional vector representations ℝ*^L^* *^×^* *^d^* are subsequently leveraged as prompt embeddings, enabling effective adaptation of the PLM for anxiety detection. Here, *d* = 768 denotes the embedding dimension and *L* represents the input sequence length, which is later used to configure different prompt lengths as part of the design.

### 3.4 Prompt-based framework

The proposed prompt-based framework, *SOFIA*, adapts the vanilla contextualized embeddings for downstream anxiety detection by integrating three key subcomponents: the prompt template *T*, the prompt verbalizer *V*, and the prompt executor *G*. These subcomponents work together to reformulate input text, map predictions to anxiety-specific labels, and efficiently utilize the frozen PLM, thereby enabling task adaptation without fine-tuning.

#### 3.4.1 Prompt template

The prompt template, shown as ① in Figure 1, reformulates the input text into a task-specific format suitable for anxiety detection. We adopt multiple template classes from the OpenPrompt library (53), including manual templates, soft templates, and mixed templates, to encapsulate the input for downstream tasks. Formally, given an input post $x=\{x_{1},x_{2},\;\ldots ,x_{L}\}$, a prompt template *T*(⋅) maps it into a new sequence Equation 1:

$$x^{\prime }=T(x)=\{\left[CLS],t_{1},\ldots ,t_{m},x_{1},x_{2},\ldots ,x_{L},t_{m+1},\ldots ,t_{m+k},[SEP\right]\}\;$$ (1)

where $\left\{t_{1},\;\ldots ,{t_{m}}_{+k}\right\}$ are template tokens. In manual templates, these tokens are predefined natural language phrases; in soft templates, they are learnable continuous embeddings; and in mixed templates, both types are combined. For example, given the anxious post, “*I can't stop overthinking everything*”, a manual template could be instantiated as:


$$x^{\prime }="[CLS]\;I\;can{}^{\prime }t\;stop\;overthinking\;everything.\;This\;text\;indicates\;[MASK]\;anxiety.\;[SEP]".$$

Here, the placeholder [MASK] is reserved for the PLM to generate a prediction token representing the underlying label, which will later be mapped into the final anxiety classes through the verbalizer. Soft and mixed templates extend this formulation by replacing parts of the natural language tokens with soft learnable embeddings, thereby allowing richer contextual adaptation while maintaining interpretability (18). The length of (⋅) is also treated as a design consideration during evaluations, as varying prompt lengths can influence contextualization and classification performance. Through this design, the template serves as the first step in aligning the frozen PLM with the downstream anxiety detection task by embedding task-specific cues directly into the input representation.

#### 3.4.2 Prompt verbalizer

The prompt verbalizer, shown as ② in Figure 1, defines the mapping function V: Y→C, where *Y* is the label space and *C* is the set of candidate label words. In the proposed framework, we incorporate multiple verbalizer classes from the OpenPrompt library, namely manual verbalizer, soft verbalizer, and automatic verbalizer, to ensure flexibility and robustness (54), while providing the knowledge base for enhancing downstream reasoning. For instance, in the anxious example “*I can't stop overthinking everything*”, a manual verbalizer may specify the mapping:

V(Anxious)={anxious","worried","stressed","nervous"},

V(Non−Anxious)={"calm","relaxed","fine","okay"}.

The knowledge base-driven verbalizer is not equivalent to a conventional label-word mapping used in standard prompt verbalizers. Rather than assigning fixed label tokens, it operates as a structured, domain-informed constraint mechanism that expands and grounds label semantics using an external anxiety lexicon (55). A curated set of anxious and non-anxious terms forms this knowledge base, enabling semantically enriched, clinically informed predictions. Given the contextualized representation of the [MASK] token, ℳ computes the probability distribution over the vocabulary (Equation 2):

$$P(c|h_{\left[MASK\right]})=Softmax(W.h_{\left[MASK\right]}+b),$$ (2)

where $h_{\left[MASK\right]}$ denotes the hidden state at the masked position, W and b are PLM parameters. The verbalizer then aggregates the probabilities of the c∈C for each class y (Equation 3):

$$P(y|x)=\underset{c\in V(y)}{\sum }P(c|h_{\left[MASK\right]}).$$ (3)

To enhance expressiveness and robustness, different verbalizer strategies are employed. Manual verbalizers rely on human-defined label words (as shown earlier), which provide interpretability but are inherently limited by manual design choices. Soft verbalizers instead learn continuous embeddings for each class label, enabling flexible adaptation beyond the constraints of discrete vocabulary. Finally, automatic verbalizers discover candidate label words directly from data in a data-driven manner, thereby reducing human bias in label-word selection and improving generalization across unseen contexts. By combining these strategies with prompt templates, the framework leverages both model reasoning and data-driven adaptability for effective anxiety detection.

#### 3.4.3 Prompt executor

The prompt executor *G*, shown as ③ in Figure 1, efficiently utilizes the frozen PLM for task adaptation without requiring fine-tuning. To ensure that the most informative signals are preserved during prompting, we introduce a novel dual-end truncation strategy. Let the input sequence be $x=\{x_{1},x_{2},\;\ldots ,x_{L}\}$ with contextual embeddings $E=\{\;e_{1},\;e_{2},\;\ldots ,\;e_{L}\}$. Unlike standard single-end truncation, our dual-end strategy selectively preserves both leading contextual content and trailing self-disclosure cues while protecting prompt template tokens, ensuring critical emotional markers are retained within the model's maximum sequence length for reliable mental health screening. Formally, for a source input length *L_s_* and a prompt length *L_p_*, the effective truncated input is:

$$\left\{x_{1},\ldots ,x_{L_{s}/2},x_{L-L_{s}/2+1},x_{L},{\left[T(x)\right]}_{1},\ldots ,{\left[T(x)\right]}_{L_{p}}\right\}$$

The dual-end truncation method, shown in Algorithm 1, operates in several stages. First, the algorithm safely handles cases where the list of *shortenable tokens* $Q_{t}$ is empty, in which case the original sequence is retained. If the input length *L* does not exceed the predefined source length $L_{s}$, truncation is not applied, thereby avoiding unnecessary processing and division-by-zero errors. When truncation is required, the removable portion $K_{t}$ of the sequence is divided equally across both ends, with rounding logic applied for odd-length cases to maintain balance (Equation 4).

$$K_{t}=\frac{L_{t}}{\sum_{j=1}^{n}L_{j}}.K$$ (4)

where K is the total number of removable tokens, $L_{t}$ is the length of the $t^{\mathrm{th}}$ shortenable segment, $L_{j}$ denotes the length of the $j^{\mathrm{th}}$ shortenable segment, *n* is the number of shortenable segments, and $K_{t}$ is the number of tokens allocated for truncation from segment *t*. The calculated tokens are truncated *S_t_* while preserving the head and tail segments, where the most informative contextual cues often reside, with an additional filtering step to prevent the sequence from becoming empty.

Algorithm 1 **Dual-end Truncation.**

Input: contextual embeddings E, maximum source length $L_{s}$

Output: truncated sequence $S_{t}$

compute input length *L*

if *L* ≤ $L_{s}$ then

return E

end if

compute total removable tokens $K\;=\;L-L_{s}$

identify shortenable template segments $Q_{t}$

if $Q_{t}$ is empty then

return E

end if

compute the length $L_{t}\;$of each shortenable segment ▸ allocate removable tokens using Equation 4

round $K_{t}\;$to obtain integer token allocations

for shortenable segment do

remove allocated tokens while preserving both the beginning and end of the segment

end for

remove empty segments if any

return $S_{t}$

This formulation ensures that the preserved tokens align with the context window constraints of 100M−300M-parameter PLMs while allocating sufficient budget for prompt tokens. Mid-sized PLMs are deliberately chosen due to computational constraints and to support efficient knowledge transfer, while avoiding the prohibitive costs and very low interpretability often associated with large-scale models.

The truncated and augmented sequences are then managed through the *PromptDataLoader* class in OpenPrompt, which maintains the fundamentals of prompt tuning for effective knowledge transfer. The resulting contextual representations are processed through a non-linear transformation (⋅), followed by normalization to stabilize optimization:

$${e^{\prime }}_{i}=\frac{f(e_{i})}{{\left|\left|f(e_{i})\right|\right|}_{2}},i\in \{1,\ldots ,L_{s}+L_{\;p}\}.$$ (5)

The normalized representation $E^{\prime }$ is then passed to the downstream classifier, completing the prompt-based execution pipeline.

### 3.5 Downstream task formation

In the final stage of the proposed methodology, the *PromptForClassification* class in the OpenPrompt library integrates the previously defined subcomponents, prompt templates, verbalizers, and the prompt executor, into a unified framework for binary anxiety classification. The optimization objective is defined as the binary cross-entropy loss:

$$L=-\frac{1}{N}\overset{N}{\underset{i=1}{\sum }}[y_{i}\log{y^{\prime }}_{i}+(1-y_{i})log(1-{y^{\prime }}_{i})],$$ (6)

where *N* is the number of samples, $y_{i}\;$ is the ground truth anxiety label, and $y_{i}^{\prime }=P(y=1|x_{i};\theta )\;$ is the predicted probability of being anxious.

## 4 Experiments

We conducted experiments to answer the following research questions.

**RQ1:** How effectively does the proposed framework leverage prompt-based methods to adapt PLMs for binary anxiety detection compared to vanilla prompt-tuning approaches?
**RQ2:** What is the impact of different prompt design choices and the novel dual-end truncation method on the performance and robustness of anxiety classification across benchmark datasets?
**RQ3:** To what extent does integrating the knowledge base through multiple types of verbalizers enhance the reasoning behind the model's predictions?

### 4.1 Datasets

As shown in Table 1, three benchmark datasets of self-reported anxiety posts from Reddit and Twitter are employed for downstream evaluation. The Guo et al. corpus (56) and the Self-reported Mental Health Diagnoses (SMHD) dataset (57), released by Georgetown University under a data usage agreement (DUA), are utilized with a particular focus on user-level annotations, enabling the framework to capture linguistic patterns associated with anxiety across a user's collective posts. In contrast, the dataset introduced by Kim et al. (58) provides post-level annotations, allowing the analysis of anxiety signals within individual social media contexts.

**Table 1:** Statistical summary of users and posts.

Benchmarks	# User		# Post		Train	Validation	Test
	Anxious	Non-Anxious	Anxious	Non-Anxious			
Guo Corpus 2022	1,800	1,800	356,242	355,019	497,883	106,689	106,689
SMHD Corpus 2018	560	590	90,580	91,120	127,190	27,255	27,255
Kim Corpus 2020	49,735	27,177	39,988	39,373	55,561	11,900	11,900

Our study constitutes a retrospective secondary analysis of these established benchmarks; thus, sample sizes, inclusion criteria, and user-disjoint data splits follow the original dataset construction protocols. In the Guo corpus, posts were pruned to retain only pre-diagnosis content to reduce behavioral confounds, whereas SMHD includes treatment and matched control users as defined by its authors. None of the released datasets contain explicit sociodemographic attributes (e.g., age, gender, ethnicity), precluding demographic subgroup or fairness analysis in the present work. This limitation stems from the original dataset construction.

The NearMiss undersampling technique (59) was applied to the Kim corpus to balance the number of anxious and non-anxious posts, ensuring overall data consistency by selecting 39,988 out of 86,243 anxious posts. Additionally, the NRC WorryWords lexicon (55), a collection of over 44,000 words with real-valued scores representing their associations with anxiety, is utilized to construct the knowledge base, enriching the framework with domain-specific linguistic cues that capture anxious expressions and emotional intensity.

### 4.2 Baselines

To evaluate the proposed framework, we benchmarked it against a set of widely used PLMs comprising 100M−300M parameters. These models span both general-domain and mental health-specific variants, enabling a comprehensive comparison of linguistic and clinical representational capabilities. The evaluated models are as follows:

- BERT (60) - A bidirectional transformer model pre-trained on large-scale English corpora such as BooksCorpus and Wikipedia. It serves as a strong general-purpose baseline for contextualized text understanding, particularly in question answering and language inference.
- DistilBERT (61) - A distilled, lighter version of BERT that retains 97% of its performance while being faster and smaller, suitable for assessing efficiency-performance trade-offs.
- RoBERTa (62) - A robustly optimized variant of BERT trained with larger data and longer sequences, offering improved generalization on various downstream tasks.
- DisorBERT (16) - A disorder-oriented transformer pre-trained on large-scale Reddit posts related to mental health disorders such as anorexia, depression, and self-harm, designed to better capture symptom expression patterns and linguistic nuances of online self-disclosure.
- MentalBERT (14) - A BERT-based model pre-trained on a curated corpus of Reddit mental health communities, enabling it to better understand psychological discourse and context-sensitive expressions of distress, anorexia, and suicidal ideation for downstream tasks.
- PsychBERT (15) - A psychology-informed, explainability-infused model trained on both clinical and social media datasets, integrating psycholinguistic features to enhance the recognition of emotional and mental health signals for depression and suicidal thoughts screening.

We intentionally limited our evaluation to 100M−300M-parameter pre-trained models for examining efficient knowledge transfer within the proposed framework, while avoiding the prohibitive computational costs and very low interpretability often associated with large-scale PLMs containing billions of parameters.

### 4.3 Implementation details

The proposed methods for anxiety screening were implemented using Python, PyTorch 2.0, and the Hugging Face Transformers^1^ libraries. The OpenPrompt framework was utilized to implement the prompt-based architecture, where the ManualTemplate, SoftTemplate, and MixedTemplate classes were employed for prompt template construction, and the ManualVerbalizer, SoftVerbalizer, and AutomaticVerbalizer classes were used for verbalizer implementation. To accommodate the model context window, the maximum input source length was adjusted in conjunction with prompt lengths (*l*) of 10, 20, and 30, ensuring that the combined prompt and text input remained within each model's context length limit. For downstream optimization, we adopted GELU activations (63) for non-linear transformations and the Adam optimizer (64) with a learning rate of 0.005 and a warmup proportion of 0.1. All models were trained for 10 epochs, with training a single model requiring approximately 39–48 h on an NVIDIA RTX 3060 Ti GPU (8 GB VRAM) with a batch size of 16. All PLMs were evaluated using identical data splits and hyperparameters to ensure fairness, and the open-source code promotes reproducibility and future research advancements^2^.

### 4.4 Metrics and results

We used precision, recall, and F1 score to evaluate downstream anxiety classification. Among these metrics, F1 score was prioritized because it jointly captures precision and recall, both of which are critical in mental health screening where false positives and false negatives carry meaningful consequences. This choice is consistent with prior anxiety screening studies, where F1 is widely adopted as the primary measure of downstream performance (57, 58). Table 2 shows evaluation results using random seed 42 for the proposed prompt-based methods and prompt-tuning baselines on binary anxiety classification. Mean ± SD across three random seeds are reported in Supplementary Appendix D. Here, we primarily employed soft templates and verbalizers, as similar configurations have demonstrated superior semantic alignment in previous mental health studies (35, 65). To quantify uncertainty, 95% confidence intervals for the top-performing models were estimated using 2,500 non-parametric bootstrap resamples of the full test sets and are reported in Supplementary Appendix A.

**Table 2:** Evaluation results of anxiety classification in terms of F1 score (%).

Setting	Model	Guo Corpus			SMHD Corpus			Kim Corpus		
		*l* = 10	*l* = 20	*l* = 30	*l* = 10	*l* = 20	*l* = 30	*l* = 10	*l* = 20	*l* = 30
Vanilla prompt-tuning methods	BERT	47.08	52.16	50.83	69.01	70.34	69.47	66.59	71.67	69.31
	DistilBERT	40.24	44.57	42.26	56.67	58.09	57.06	54.24	59.64	57.63
	RoBERTa	43.06	48.61	45.37	60.36	62.83	61.17	68.06	73.35	71.04
	DisorBERT	39.58	42.13	41.05	68.63	69.82	69.33	69.64	73.82	72.08
	PsychBERT	52.93	56.26	54.04	68.17	69.07	68.35	74.31	78.23	76.27
	MentalBERT	53.36	58.17	55.24	70.22	71.84	71.53	75.06	79.81	78.07
Proposed prompt-based methods	BERT	52.03	57.64	54.08	75.90	75.93	74.87	76.38	81.57	78.62
	DistilBERT	44.73	49.76	46.81	61.23	61.80	61.56	58.30	63.64	60.24
	RoBERTa	45.19	52.56	47.58	65.76	66.88	65.92	79.83	83.87	81.62
	DisorBERT	44.73	48.51	46.27	74.38	75.72	74.81	80.25	84.36	82.18
	PsychBERT	57.05	61.27	59.05	74.79	75.54	75.24	83.95	87.08	85.63
	MentalBERT	58.02	**63.54**	60.34	76.19	**77.24**	77.03	84.06	**87.73**	86.71

Best performance is shown in bold.

### 4.5 Ablation studies

To assess the contribution of individual design components within the proposed framework, we conducted a series of ablation experiments focusing on prompt design and dual-end truncation, addressing RQ2. Specifically, we evaluated the effect of different prompt template types with a fixed template length of 20, both with and without the dual-end truncation method, across the Guo and Kim corpora. The Guo corpus enables evaluation of model consistency and generalization across individual users while the Kim corpus allows finer-grained assessment of contextual adaptability at the utterance level. The results, shown in Table 3, illustrate the top three PLMs in terms of F1 score, highlighting how the proposed truncation strategy enhances contextual retention and robustness.

**Table 3:** Results of the ablation study on the effects of different prompt template types (*l* = 20) and truncation methods in terms of F1 score (%).

Setting	Model	Guo Corpus			Kim Corpus		
		Manual	Soft	Mixed	Manual	Soft	Mixed
With single-end truncation	BERT	49.61	54.27	51.34	71.38	77.63	73.64
	PsychBERT	52.36	58.91	55.63	75.59	83.59	79.03
	MentalBERT	54.69	60.08	57.18	76.08	84.28	79.84
With dual-end truncation	BERT	52.58	57.64	54.85	76.24	81.57	78.51
	PsychBERT	55.83	61.27	58.03	78.05	87.08	83.18
	MentalBERT	58.07	**63.54**	60.29	79.36	**87.73**	84.24

Best performance is shown in bold.

**Figure 2:**
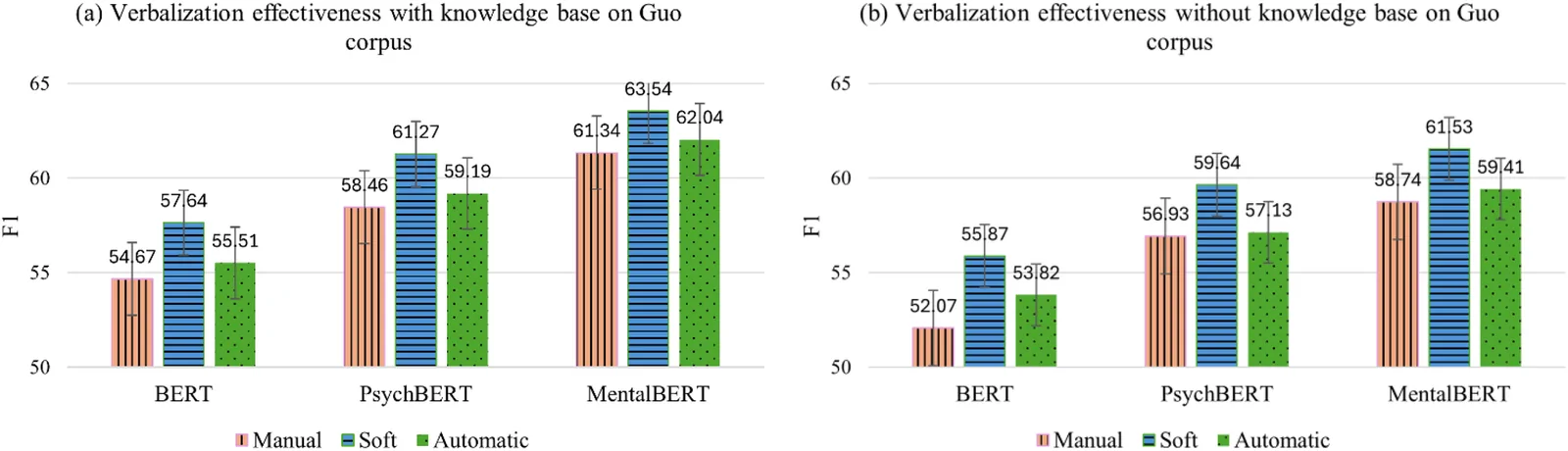
Verbalization effectiveness with and without knowledge base on Guo corpus in terms of F1 score (%).

**Figure 3:**
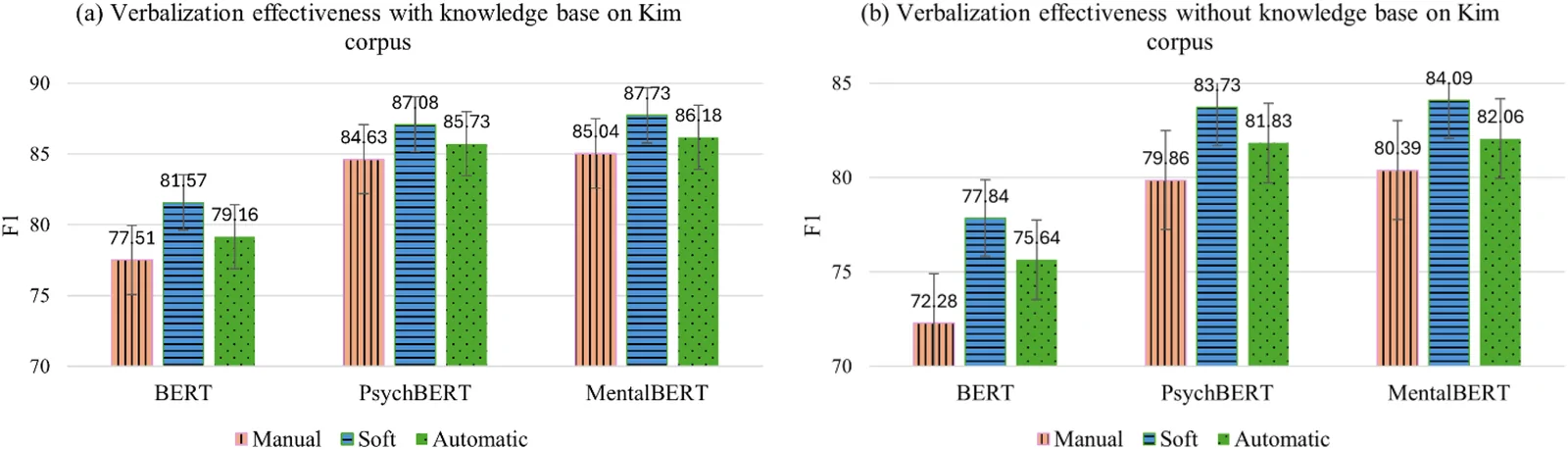
Verbalization effectiveness with and without knowledge base on Kim corpus in terms of F1 score (%).

Furthermore, to address RQ3, we examined the impact of integrating the knowledge base through multiple types of verbalizers with a template length of 20. This analysis explores how verbalizer selection influences knowledge-guided model inference and knowledge transfer efficiency. Figures 2,3 presents the comparative performance of the top three PLMs on both Guo and Kim corpora, demonstrating the effectiveness of knowledge-enhanced verbalization.

### 4.6 Statistical test

To assess whether the observed performance gains are statistically reliable, we conducted significance testing using McNemar's test, which is well-suited for paired comparisons in binary classification. McNemar's test evaluates whether two models exhibit significantly different instance-level error patterns when evaluated on the same test set. This makes it particularly suitable for our controlled comparisons of prompt-based anxiety classifiers, where predictions are generated for identical instances under different modeling strategies. Moreover, since the task is formulated as binary anxiety screening, McNemar's test provides a standard and robust non-parametric approach without requiring distributional assumptions.

We performed statistical testing on 2,500 test set samples from the Guo corpus (user-level annotations) and the Kim corpus (post-level annotations) to reflect both coarse-grained and fine-grained screening conditions. McNemar's test is applied to compare: (a) vanilla prompt-tuning baselines vs. the proposed prompt-based methods and (b) dual-end truncation vs. standard single-end truncation. Detailed McNemar's test results, including the McNemar test statistic (*χ*²) and corresponding *p*-values under the chi-square distribution with one degree of freedom, are reported in Supplementary Appendix B.

## 5 Discussion

### 5.1 Performance on prompt-based anxiety screening

The experimental results on anxiety screening demonstrate that the proposed prompt-based framework substantially improves the adaptation of PLMs to previously unseen anxiety classification tasks. By introducing a task-driven redesign of prompt-based transfer, including soft prompt templates, knowledge base-driven verbalizers grounded in clinically informed lexical resources, and the proposed dual-end truncation strategy that preserves both early contextual cues and late-stage emotional markers, the framework enables more robust and efficient knowledge transfer from both general-purpose and mental health-specific PLMs without requiring traditional prompt-tuning. As shown in Table 2, MentalBERT achieved the best overall performance across all benchmark datasets, outperforming other 100M−300M-parameter, vanilla prompt-tuning baseline PLMs for anxiety detection.

Across the Guo corpus, which features user-level annotations, MentalBERT, PsychBERT, and BERT exhibited consistent improvements in F1 score, 5.37%, 5.01%, and 5.48%, respectively, over baseline prompt-tuning techniques when using soft templates of length (*l*) 20 and knowledge base-driven soft verbalizers. McNemar's test with continuity correction showed that *SOFIA* significantly outperformed the vanilla prompt-tuning baseline for BERT (*χ*² = 8.05, *p* = 0.0045), DistilBERT (*χ*² = 5.65, *p* = 0.0174), PsychBERT (*χ*² = 7.11, *p* = 0.0077), and MentalBERT (*χ*² = 6.18, *p* = 0.0130), while improvements for RoBERTa and DisorBERT were not statistically significant (*p* > 0.05). These results suggest that the proposed framework is associated with improved task performance across several PLMs, potentially reflecting more effective alignment between general linguistic knowledge and mental health-specific representations in the anxiety screening task. The incorporation of the knowledge base may contribute to more structured label grounding; however, this interpretation should be considered in light of the non-interventional study design. Template length (*l*) also influenced performance. Increasing *l* from 10 to 20 tokens improved performance across all PLMs by enabling richer contextual representations. This effect is attributed to the proposed dual-end truncation method, which ensures that critical segments from both the beginning and end of each input sequence are preserved. These regions frequently encode key emotional and cognitive signals necessary for accurate anxiety classification. However, performance slightly decreased when *l* reached 30, suggesting a saturation effect where overly long templates may dilute the relevance of prompt tokens and increase task ambiguity, particularly under the parameter constraints of the 100M−300M-parameter PLMs used in this study, which limit their capacity to effectively model extended contextual dependencies.

A similar trend was observed for the SMHD corpus, where MentalBERT again achieved the highest performance among mid-sized PLMs. This relative advantage may be related to its pre-training on mental health-relevant Reddit data, which includes context-sensitive expressions associated with distress and related conditions. However, the F1 score improvements observed when increasing *l* from 10 to 20 were smaller in SMHD than in the Guo corpus. This difference may reflect the shorter average post length in SMHD, which potentially limits the added benefit of longer templates and extended contextual conditioning. For the Kim corpus, which provides post-level annotations, comparatively larger performance gains were observed, indicating that the framework generalizes across annotation granularities. MentalBERT achieved an F1 score of 87.73%, marking an improvement of 7.92% over vanilla prompt-tuned MentalBERT. The post-level structure enables models to capture utterance-level anxiety cues, improving the detection of subtle linguistic patterns that may be lost in user-level aggregation. Repeated experiments with multiple random seeds demonstrated stable and consistent performance, particularly for the proposed methods, which exhibited a lower standard deviation. PsychBERT and DisorBERT showed the largest relative F1 improvements (8.85% and 10.54%, respectively), with PsychBERT achieving statistically significant gains, whereas DistilBERT's improvement did not reach statistical significance. Specifically, continuity-corrected McNemar's tests on the Kim test set indicated that *SOFIA* significantly outperformed the vanilla prompt-tuning baseline across all evaluated PLMs, including BERT (*χ*² = 48.94, *p* < 0.0001), DistilBERT (*χ*² = 6.07, *p* = 0.0138), RoBERTa (*χ*² = 14.73, *p* = 0.00012), DisorBERT (*χ*² = 20.15, *p* < 0.0001), PsychBERT (*χ*² = 68.88, *p* < 0.0001), and MentalBERT (*χ*² = 80.01, *p* < 0.0001). These results confirm that the observed performance gains are statistically reliable and reflect consistent reductions in instance-level prediction errors relative to the vanilla prompt-tuning baselines. While the dual-end truncation method ensured contextual preservation, the longer sequences in Kim's dataset increased sensitivity to template length. Yet, top-performing models consistently benefited from combining soft prompts and soft verbalizers, emphasizing the synergy between flexible contextual learning and adaptive label grounding.

To further assess the robustness of the observed performance gains, we computed 95% confidence intervals for the F1-score differences between vanilla prompt tuning and *SOFIA* using non-parametric bootstrap resampling. On the Guo corpus, *SOFIA* improved the F1 score by 5.48 percentage points for BERT [95% CI: (4.1, 7.2)], 5.01 points for PsychBERT [95% CI: (2.8, 5.7)], and 5.37 points for MentalBERT [95% CI: (3.2, 5.8)]. On the Kim corpus, *SOFIA* achieved larger improvements of 9.90 points for BERT [95% CI: (9.2, 13.4)], 8.85 points for PsychBERT [95% CI: (8.4, 13.1)], and 7.92 points for MentalBERT [95% CI: (6.8, 10.7)]. Notably, all confidence intervals exclude zero, indicating that the observed improvements are consistently positive and statistically reliable across both corpora. While these results demonstrate statistically reliable performance improvements under controlled benchmark settings, they should not be interpreted as evidence of direct clinical effectiveness. Establishing real-world bedside applicability would require prospective validation, clinician-in-the-loop evaluation, and assessment within diverse patient populations, which are beyond the scope of the present study.

Overall, the proposed prompt-based methods enable efficient knowledge adaptation in mid-sized PLMs, offering a computationally economical alternative to traditional prompt-tuning. The results collectively demonstrate that MentalBERT, PsychBERT, and BERT are the top three performing PLMs for the downstream anxiety classification task across all benchmark datasets. Furthermore, this study represents the first systematic attempt to explore prompt-based anxiety detection, addressing a previously unexamined gap in mental health NLP research, and establishes a foundation for cost-effective and reasoning-aware PLM adaptation. Therefore, RQ1: *How effectively does the proposed framework leverage prompt-based methods to adapt PLMs for binary anxiety detection compared to vanilla prompt-tuning approaches?* can be conclusively answered.

### 5.2 Impact of prompt template types and truncation

To investigate the role of different prompt template designs and the proposed dual-end truncation method, we conducted an ablation study focusing on prompt length 20, as shown in Table 3. The analysis aims to ensure that the most contextually relevant and emotionally informative input signals are preserved for effective prompting within the model's context window. Across the top three PLMs, soft prompt templates yielded higher F1 scores than mixed and manual templates, suggesting greater flexibility in learning task-relevant representations within a continuous embedding space. Mixed templates demonstrated intermediate performance, whereas manual templates showed comparatively lower results, possibly reflecting their fixed, human-defined structure. Among the mid-sized PLMs, MentalBERT achieved the highest performance on the Guo corpus, with a 5.9% F1 score improvement when using the dual-end truncation strategy relative to the baseline configuration. Compared with single-end truncation, dual-end truncation was associated with improvements of up to 3.46% in F1 score. These findings suggest that preserving both initial and final segments of the input text may provide additional contextual cues relevant to anxiety classification. Importantly, these performance gains are statistically robust. Continuity-corrected McNemar's tests on the Guo test set demonstrated that dual-end truncation significantly outperformed single-end truncation for BERT (*χ*² = 22.28, *p* < 0.0001), PsychBERT (*χ*² = 18.87, *p* < 0.0001), and MentalBERT (*χ*² = 18.01, *p* < 0.0001). The magnitude of the *χ*² statistics reflects consistent reductions in instance-level disagreements between configurations; however, these results should be interpreted within the constraints of the benchmark-based evaluation setting.

The performance further improved on the Kim corpus, where MentalBERT achieved the highest F1 score of 87.73%, surpassing PsychBERT by a marginal 0.65% employing soft templates. This improvement likely stems from the post-level annotations in Kim's dataset, which capture finer-grained psycholinguistic cues. Once again, the proposed truncation strategy effectively preserved both initial contextual cues and concluding emotional markers, providing richer signals for soft prompt optimization and achieving a maximum F1 score gain of 3.45% compared to standard single-end truncation. On the Kim test set, continuity-corrected McNemar's tests indicated that dual-end truncation significantly outperformed standard single-end truncation for PsychBERT (*χ*² = 4.22, *p* = 0.040) and MentalBERT (*χ*² = 4.00, *p* = 0.046). Although BERT exhibited a similar trend, the improvement did not reach statistical significance (*χ*² = 2.74, *p* = 0.098). Overall, the findings confirm that the proposed dual-end truncation combined with soft prompting enhances contextual retention, robustness, and emotional sensitivity across datasets. Therefore, RQ2: *What is the impact of different prompt design choices and the novel dual-end truncation method on the performance and robustness of anxiety classification across benchmark datasets?* can be considered answered, validating the methodological contributions of the framework.

### 5.3 Impact of knowledge base across verbalizer types

To further assess the influence of the proposed knowledge base on reasoning, an ablation study was conducted using multiple verbalizer configurations, as shown in Figures 2,3. This experiment examines how integrating the knowledge base relates to the performance of manual, soft, and automatic verbalizers across benchmark anxiety datasets. Consistent with the findings observed for different prompt template types, the soft verbalizer achieved the highest performance, outperforming both manual and automatic verbalizers with and without the knowledge base. These results suggest that soft verbalizers may facilitate more flexible class representations within a continuous embedding space, potentially strengthening associations between label tokens and task-relevant linguistic features. Automatic verbalizers achieved moderate performance, benefiting from reduced human bias in label-word selection and improved generalization, while manual verbalizers showed the lowest performance due to limited adaptability and rigid label mapping.

According to Figure 2B, removing the knowledge base resulted in a measurable decline in model performance across the Guo corpus, with F1 score reductions of 2.01%, 1.63%, and 1.77% for MentalBERT, PsychBERT, and BERT, respectively. This degradation highlights the importance of the knowledge base in maintaining high-quality *anxious* and *non-anxious* label associations and in strengthening the PLMs' reasoning capabilities during prompt-driven inference. The absence of the knowledge base led to reduced conceptual grounding for anxiety-related cues, reflected by lower consistency in the generated predictions. A similar yet more pronounced trend was observed on the Kim corpus, which features post-level annotations capturing fine-grained contextual and emotional expressions. As shown in Figure 3B, removing the knowledge base led to larger declines in F1 score, 3.64%, 3.35%, and 3.73% for MentalBERT, PsychBERT, and BERT, respectively. This more pronounced reduction suggests that the knowledge base may contribute to performance in post-level prediction settings, where richer linguistic cues are often present. However, this interpretation should be considered within the constraints of the benchmark-based evaluation framework.

Overall, these results confirm that integrating the knowledge base with soft verbalizers substantially improves knowledge-guided model inference and knowledge transfer efficiency across multiple datasets. Therefore, RQ3: *To what extent does integrating the knowledge base through multiple types of verbalizers enhance the reasoning behind the model's predictions?* can be conclusively addressed.

### 5.4 Challenges and limitations

One of the foremost challenges encountered in this study was the scarcity of self-reported social media data explicitly related to anxiety. Unlike depression or suicidal ideation datasets, anxiety-related corpora are relatively limited in both quantity and annotation quality, constraining the robustness of model evaluation and generalization. Another key challenge involved ensuring adequate data anonymization while maintaining the contextual integrity of posts. Preserving linguistic cues essential for anxiety detection, such as subtle emotional markers, proved difficult when personally identifiable or sensitive information needed to be removed. The design and selection of prompt templates, verbalizers, and knowledge bases also introduced potential biases. Manual construction of these components may implicitly reflect subjective assumptions regarding how anxiety is expressed linguistically. In addition, effectively integrating the OpenPrompt framework required overcoming implementation challenges related to properly utilizing the *Template* and *Verbalizer* classes. Specifically, aligning handcrafted and knowledge base-driven verbalizers with model tokenization schemes demanded extensive experimentation to achieve stable convergence. Another practical consideration was managing annotation granularity. The framework relied on both post-level and user-level annotations across corpora, each presenting unique advantages and trade-offs. Post-level annotations enabled fine-grained contextual interpretation but limited broader behavioral insights. User-level annotations captured user-centric patterns but sacrificed contextual specificity. Balancing both perspectives was essential to evaluate the proposed approach's adaptability.

The study presents several notable limitations. First, the experiments were restricted to a dual-end truncation strategy within OpenPrompt, and other generic truncation or context selection techniques, including head/tail truncation or dynamic attention pruning (66), were not explored. Second, although the framework performed effectively on 100M−300M-parameter PLMs within a prompt-based adaptation setting, further evaluation with larger-scale models as well as comparisons against fully fine-tuned counterparts, are needed to verify scalability and assess whether larger parameter capacities can better capture long-range contextual dependencies. Third, this work focused exclusively on binary anxiety classification as the downstream task. Extending the framework to other mental health conditions, such as depression, PTSD, or comorbid states, would provide broader validation of its generalizability. Moreover, the limited context window of the PLMs constrained the capacity to encode longer discourse segments, a key limitation when modeling multi-turn conversations or extended user histories. Another key limitation of this study is the absence of demographic metadata in the publicly released datasets, which prevents subgroup-based fairness and bias analyses. As anxiety-related language may vary across demographic, cultural, and linguistic groups, the generalizability of the proposed framework to diverse populations cannot be fully established. Future work should evaluate the framework on ethically sourced datasets containing demographic annotations to enable comprehensive fairness assessment and validate its robustness across diverse populations and clinical settings. The current reasoning mechanism is primarily driven by knowledge base-informed verbalizers within a prompt-based classification setup. While this design enhances structured label alignment, it does not incorporate more advanced reasoning paradigms, such as chain-of-thought prompting or reason-then-predict strategies, which may further improve interpretability and decision transparency, particularly when scaling to larger PLMs. Additionally, because the datasets are derived from English-language Reddit and Twitter users, transferability to other regions, cultural contexts, languages, and healthcare systems remains uncertain. Temporal drift in language use across platforms may also affect model robustness over time. External validation on independent datasets and within real-world clinical workflows is necessary to confirm stability, reproducibility, and practical utility beyond benchmark settings. Finally, the proposed framework is a screening tool intended for early detection and digital triage, and should not be interpreted as a standalone diagnostic or clinical decision-making system.

From an ethical standpoint, several aspects merit close attention. This study constitutes a retrospective secondary analysis of publicly available benchmark datasets; therefore, sample sizes, inclusion criteria, and data splits strictly follow the original dataset construction protocols. We adhered to the data usage agreements and IRB frameworks under which these datasets were originally collected and released. No attempt was made to re-identify users, link accounts across platforms, or infer hidden identities. All analyses were conducted on anonymized data in accordance with the ethical standards specified by the source institutions. Nevertheless, models trained on self-reported social media data present inherent ethical challenges. Although posts are publicly accessible, mental health disclosures remain highly sensitive, and automated modeling may risk inadvertent privacy violations or inference of protected attributes. None of the released datasets contain explicit sociodemographic metadata, which limits fairness auditing but also reflects privacy-preserving design choices by the original authors. To mitigate data security risks, we implemented encrypted storage, restricted access controls, and privacy-compliant PLM hosting environments throughout experimentation. Bias and interpretability also require careful consideration. The pre-trained knowledge embedded within PLMs may encode platform- or population-specific biases, potentially leading to unfair predictions across culturally diverse contexts. The explainability of the constructed knowledge base and verbalizer mappings remains an ongoing challenge, particularly in ensuring that candidate labels reflect domain knowledge without amplifying stereotypes. Accountability concerns additionally arise in prompt template curation, where subtle linguistic framing decisions may influence model outputs. Prompt-based mental health screening further introduces uncertainty and confidentiality considerations when mapping emotional or cognitive states to discrete labels. Importantly, the proposed framework is intended solely for research and early digital triage. Any real-world or bedside deployment would require prospective validation, clinician oversight, regulatory review, and assessment of potential psychological impact on users. Continuous monitoring, periodic bias audits, and robust governance mechanisms are therefore essential to ensure responsible and ethically aligned application.

## 6 Conclusion and future work

In this study, we propose *SOFIA* (*Anxious? SOFten-It-out*), a novel prompt-based reasoning framework designed to efficiently adapt PLMs for anxiety screening from social media posts. *SOFIA* integrates a dual-end truncation strategy with multiple prompt template types, allowing the preservation of both initial contextual cues and concluding emotional expressions that are critical for accurate anxiety detection. The framework further incorporates a knowledge base enriched with clinical domain knowledge, which enhances model reasoning while maintaining computational efficiency. Comprehensive experiments were conducted across benchmark self-reported anxiety corpora demonstrated that *SOFIA* consistently outperforms vanilla prompt-tuning baselines across all tested 100M−300M-parameter PLMs. The results, including ablation studies, confirm that the proposed methods significantly improve knowledge transfer, knowledge-guided model inference, and contextual adaptability in mental health-related text understanding.

Future research may explore scaling the framework to larger models with more than 1B parameters and comparing it against fully fine-tuned counterparts to investigate scalability, performance-efficiency trade-offs, and reasoning consistency. Additionally, integrating alternative truncation and sampling strategies could further improve context preservation and reduce information loss. Expanding *SOFIA* beyond anxiety detection to multi-class mental health screening offers promising potential. Moreover, future studies should investigate bias mitigation and fairness in knowledge base construction and verbalizer generation, while also evaluating clinically relevant measures such as positive predictive value under realistic prevalence settings to assess real-world screening utility. Finally, integrating explainable AI techniques and cross-lingual evaluations may enhance transparency and global applicability, advancing *SOFIA* as a reliable tool for early digital mental health support.

## Data Availability

The original contributions presented in the study are included in the article/Supplementary Material, further inquiries can be directed to the corresponding author.

## Footnotes

^1^ https://github.com/huggingface/transformers

^2^Code will be published upon acceptance.
