# Supplementary material for: SOFIA: a knowledge-infused prompt-based reasoning framework for anxiety screening in social media
Source: Front Digit Health. 2026 Aug 14;8:1899582. doi: 10.3389/fdgth.2026.1899582 (PMC13521909; doi:10.3389/fdgth.2026.1899582)
Supplement: Supplementary file 1 [file Supplementaryfile1.docx]

# Appendix A – F1 scores and 95% bootstrap CIs for the top models on the full test sets (*l*=20)

| Model | Guo Corpus | | | | Kim Corpus | | | |
| --- | --- | --- | --- | --- | --- | --- | --- | --- |
|  | Baseline | *SOFIA* | ΔF1 | 95% CI of ΔF1 | Baseline | *SOFIA* | ΔF1 | 95% CI of ΔF1 |
| BERT | 52.16 | 57.64 | 5.48 | [4.1, 7.2] | 71.67 | 81.57 | 9.90 | [9.2, 13.4] |
| PsychBERT | 56.26 | 61.27 | 5.01 | [2.8, 5.7] | 78.23 | 87.08 | 8.85 | [8.4, 13.1] |
| MentalBERT | 58.17 | 63.54 | 5.37 | [3.2, 5.8] | 79.81 | 87.73 | 7.92 | [6.8, 10.7] |

# Appendix B

## (a. I) McNemar’s test for vanilla prompt-tuning baselines vs. the proposed prompt-based methods on Guo test set (*N*=2,500): Table 2

|  | # baseline ✓ & *SOFIA* ✗ | # baseline ✗ & *SOFIA* ✓ | χ² | p-value |
| --- | --- | --- | --- | --- |
| BERT | 670 | 779 | 8.051 | **0.0045** |
| DistilBERT | 512 | 592 | 5.652 | **0.0174** |
| RoBERTa | 584 | 627 | 1.461 | 0.227 |
| DisorBERT | 482 | 512 | 0.849 | 0.358 |
| PsychBERT | 692 | 798 | 7.112 | **0.0077** |
| MentalBERT | 728 | 827 | 6.179 | **0.013** |

## (a. II) McNemar’s test for vanilla prompt-tuning baselines vs. the proposed prompt-based methods on Kim test set (*N*=2,500): Table 2

|  | # baseline ✓ & *SOFIA* ✗ | # baseline ✗ & *SOFIA* ✓ | χ² | p-value |
| --- | --- | --- | --- | --- |
| BERT | 287 | 482 | 48.94 | **<0.0001** |
| DistilBERT | 402 | 476 | 6.07 | 0.0138 |
| RoBERTa | 322 | 428 | 14.73 | 0.00012 |
| DisorBERT | 308 | 431 | 20.15 | **<0.0001** |
| PsychBERT | 146 | 327 | 68.88 | **<0.0001** |
| MentalBERT | 137 | 331 | 80.01 | **<0.0001** |

## (b. I) McNemar’s test for dual-end truncation vs. standard single-end truncation on Guo test set (*N*=600): Table 3

|  | # baseline ✓ & *SOFIA* ✗ | # baseline ✗ & *SOFIA* ✓ | χ² | p-value |
| --- | --- | --- | --- | --- |
| BERT | 124 | 212 | 22.28 | **0.0001** |
| PsychBERT | 137 | 223 | 18.87 | **0.0001** |
| MentalBERT | 141 | 232 | 18.01 | **0.0001** |

## (b. II) McNemar’s test for dual-end truncation vs. standard single-end truncation on Kim test set (*N*=600): Table 3

|  | # baseline ✓ & *SOFIA* ✗ | # baseline ✗ & *SOFIA* ✓ | χ² | p-value |
| --- | --- | --- | --- | --- |
| BERT | 97 | 122 | 2.74 | 0.098 |
| PsychBERT | 61 | 87 | 4.22 | **0.040** |
| MentalBERT | 53 | 78 | 4.00 | **0.046** |

# Appendix C

# (a. I) Training and validation loss curves for BERT (*left*), PsychBERT (*middle*), and MentalBERT (*right*) on the Guo corpus

# (a. II) Training and validation loss curves for BERT (*left*), PsychBERT (*middle*), and MentalBERT (*right*) on the Kim corpus

# Appendix D - Evaluation results of anxiety classification in terms of F1 score (mean ± SD over three random seeds: 42, 127, 999)

| **Setting** | **Model** | **Guo Corpus** | | | **Kim Corpus** | | |
| --- | --- | --- | --- | --- | --- | --- | --- |
|  |  | ***l*=10** | ***l*=20** | ***l*=30** | ***l*=10** | ***l*=20** | ***l*=30** |
| *Vanilla*  *prompt-tuning*  *methods* | BERT | 47.08 ± 0.23 | 52.16 ± 0.21 | 50.83 ± 0.20 | 66.59 ± 0.21 | 71.67 ± 0.20 | 69.31 ± 0.22 |
|  | PsychBERT | 52.93 ± 0.19 | 56.26 ± 0.23 | 54.04 ± 0.22 | 74.31 ± 0.26 | 78.23 ± 0.23 | 76.27 ± 0.25 |
|  | MentalBERT | 53.36 ± 0.21 | 58.17 ± 0.26 | 55.24 ± 0.24 | 75.06 ± 0.24 | 79.81 ± 0.21 | 78.07 ± 0.23 |
| *Proposed*  *prompt-based*  *methods* | BERT | 52.03 ± 0.18 | 57.64 ± 0.17 | 54.08 ± 0.19 | 76.38 ± 0.17 | 81.57 ± 0.16 | 78.62 ± 0.18 |
|  | PsychBERT | 57.05 ± 0.15 | 61.27 ± 0.15 | 59.05 ± 0.16 | 83.95 ± 0.18 | 87.08 ± 0.15 | 85.63 ± 0.17 |
|  | MentalBERT | 58.02 ± 0.16 | 63.54 ± 0.14 | 60.34 ± 0.15 | 84.06 ± 0.16 | 87.73 ± 0.13 | 86.71 ± 0.15 |
